# Consumption and Overcrowding

**Published:** 1899-01-28

**Authors:** 


### CONSUMPTION AND OVERCROWDING.

THE subjoined tables, which are taken from the Annual Report of the Medical Officer of Health for the County of London, are of considerable interest in regard to the now prominent question of the prevention of consumption. The various districts in the metropolis being arranged in groups according to the amount of overcrowding present in each, these tables show (1) the death-rates from phthisis in each group of districts; and (2) the comparative death-rates, that is the death-rate in each group compared with that in the one which is the least overcrowded the latter being expressed as 100, and all the groups being worked out to show the rates at each age period. If one reads these tables aright the results are very striking. According to the first table it will be seen that the death-rate from phthisis rises in proportion as the overcrowding of the district increases, and this not in small or insignificant proportion, but to such an extent that, while in the least overcrowded districts the death-rate from phthisis was only 1·14 (per 1,000 living), in those districts where overcrowding was most prevalent the death-rate rose to much more than double, namely, 2·64. If this had happened in one year only, or if it had been observed merely in regard to one degree of overcrowding, it would not be so significant; but it will be observed that in every year, and in regard to every degree of overcrowding, it is the case that, as the proportion of overcrowding increases, so does the death-rate from phthisis. In view, however, of the possibility that the overcrowded districts might perhaps be less favourably situated than others in regard to age constitution, Mr. Shirley Murphy had the figures worked out in regard to a series of age periods with the startling results shown in the second table, according to which it would seem as if the more prolonged the exposure to the evil influences which are associated with overcrowding the greater was the tendency to phthisis. After the first five years of life the tendency of phthisis in London is to be increasingly fatal at each age period up to the decennium 35 to 45, after which it becomes a less frequent cause of death, and an inspection of other tables given in Mr. Murphy's report shows that under all conditions of overcrowding this relation holds good. But the curve cut by these figures is vastly different in districts having different degrees of overcrowding. This is shown by taking the prevalence of phthisis at each age period in districts with less than 10 per cent. of overcrowding as the normal phthisical death-rate, and setting out the death-rates for the other ages in proportion to this standard. This table, then of comparative death-rates shows that, although at the ages 5—20 the varying degrees of overcrowding seem to have no very marked influence, the increases in the death-rates from phthisis according to each increase in the overcrowding become much more marked at each successive age period. To such an extent is this the case that the mortality from phthisis in the most closely crowded districts for people between 45 and 55 years old is more than twice, and that for those over 55 is nearly four times, as great as it is for people of similar ages living in parts where overcrowding is least prevalent. The consistency of the figures given below leads to the conclusion that the results are not accidental, and one can hardly escape the conclusion that overcrowding and the conditions of life which accompany it are potent influences in the production of consumption. The bearing of this on a good deal that is now being proposed in regard to the prevention of tuberculosis is pretty obvious.

TABLE I.—PHTHISIS, 1894—7.

| Proportion of total population living more than two in a room (in tenements of less than five rooms). | Death Rates per 1,000 living. | | | |
|---|---|---|---|---|
| | 1894. | 1895. | 1896. | 1897. |
| Districts with under | | | | |
| 10 per cent. ... ... | 1·07 | 1·18 | 1·07 | 1·14 |
| 10 to 15 per cent. ... | 1·38 | 1·49 | 1 46 | 1·42 |
| 15 " 20 " " ... | 1·57 | 1·64 | 1 61 | 1·63 |
| 20 " 25 " " ... | 1·81 | 1 83 | 1 67 | 1·75 |
| 25 " 30 " " ... | 2·11 | 2 09 | 2 06 | 2'10 |
| 30 " 35 " " ... | 2 26 | 2·42 | 2·13 | 2·32 |
| Over 35 " " ... | 2·46 | 2·66 | 2·55 | 2·64 |

TABLE II.—PHTHISIS: COMPARATIVE DEATH RATES—DEATH RATES IN LEAST OVERCROWDED GROUP AT EACH AGE PERIOD TAKEN AS 100.

| Proportion of total population living more than two in a room (in tenements of less than five rooms). | 0– | 5– | 20– | 25– | 35– | 45– | 55 and upwards |
|---|---|---|---|---|---|---|---|
| Districts with under | | | | | | | |
| 10 per cent. ... ... | 100 | 100 | 100 | 100 | 100 | 100 | 100 |
| 10 to 15 per cent. ... | 165 | 108 | 107 | 112 | 121 | 139 | 149 |
| 15 " 20 " " ... | 159 | 105 | 123 | 117 | 144 | 146 | 185 |
| 20 " 25 " " ... | 128 | 95 | 148 | 115 | 170 | 182 | 211 |
| 25 " 30 " " ... | 187 | 116 | 142 | 119 | 206 | 207 | 254 |
| 30 " 35 " " ... | 194 | 97 | 126 | 166 | 232 | 294 | 303 |
| Over 35 " " ... | 284 | 121 | 167 | 201 | 261 | 239 | 367 |

